# Acquired Dynamic Left Ventricular Outflow Tract Obstruction: A Rare Complication of Acute Myocardial Infarction

**DOI:** 10.1016/j.case.2021.12.006

**Published:** 2022-01-19

**Authors:** Kapil Rajendran, Sivaprasad Kunjukrishnanpillai, Baiju Rajan

**Affiliations:** Department of Cardiology, Government TD Medical College Alappuzha, Kerala, India

**Keywords:** Acute myocardial infarction, Dynamic left ventricular outflow tract obstruction, Beta-blockers, Hypertrophic cardiomyopathy, Revascularization

## Abstract

• Reversible outflow tract obstruction is a rare complication of myocardial infarction.
• Immediate bedside echocardiogram can help recognize this complication.
• Sigmoid-shaped septum and narrow aortoseptal angle are likely anatomic contributors.
• Tachycardia, hypertension, and basal hypercontractility are hemodynamic factors.
• Early beta-blockers and revascularization can reverse this complication.

## Graphical abstract

**Figure:**
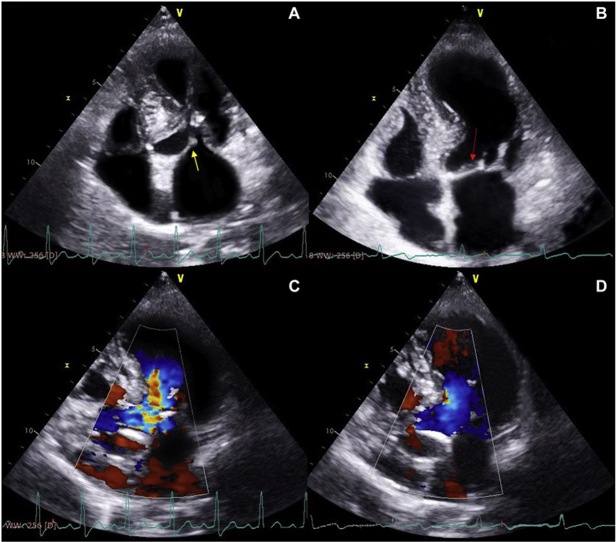

## Introduction

The mechanical complications of ST-segment elevation myocardial infarction (STEMI) include left ventricular free-wall rupture, ventricular septal rupture, acute mitral regurgitation due to rupture of chordae tendineae or papillary muscle, pseudo aneurysm, true aneurysm, cardiac tamponade, and dynamic left ventricular outflow tract (LVOT) obstruction.^1^ According to recent studies, the incidence of these mechanical complications following myocardial infarction ranges from 0.27% to 0.91%.^2^ Despite advances in pharmacological, catheter-based, and surgical reperfusion strategies that have substantially reduced the frequency of mechanical complications, these patients carry a more than fourfold increased risk for in-hospital adverse outcomes.^3^ Astute clinical recognition of these complications and emergent evaluation with echocardiography could be crucial in planning an appropriate therapeutic strategy and substantially reduce in-hospital mortality.

## Case Presentation

**Figure 1:**
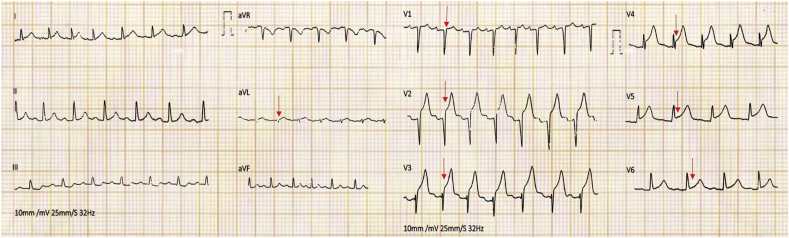
Twelve-lead electrocardiogram taken at the time of admission showing ST-segment elevation *(arrows)* in precordial leads V1-V6 and limb lead aVL.

A 75-year-old postmenopausal woman presented to the emergency department with a history of easy fatigability for a duration of 6 hours. She had a significant history of diabetes mellitus and hypertension for a duration of 10 years and was on optimal medical treatment. She had no family history of cardiac illness or sudden cardiac death. At the time of admission, her pulse rate was 112 beats per minute (bpm), with blood pressure of 140/80 mm Hg and oxygen saturation of 98% at room air. The mean pressures of her jugular venous pulse were normal, and auscultation revealed a third heart sound (S3), grade 2/6 ejection systolic murmur in the left third intercostal area, and grade 3/6 pansystolic murmur in the mitral area. Normal vesicular breath sounds were heard equally in both lung fields, and there were no basal crepitations. The patient was in Killip class II heart failure at presentation. Examinations of other systems were unremarkable. Her electrocardiogram at admission showed ST-segment elevation in lead aVL and precordial leads V2-V6 (Figure 1) suggestive of an acute anterior wall STEMI. Her blood sugar at admission was 472 mgs%, arterial blood gas analysis was negative for metabolic acidosis, and urine acetones were negative for ketone bodies.

**Figure 2:**
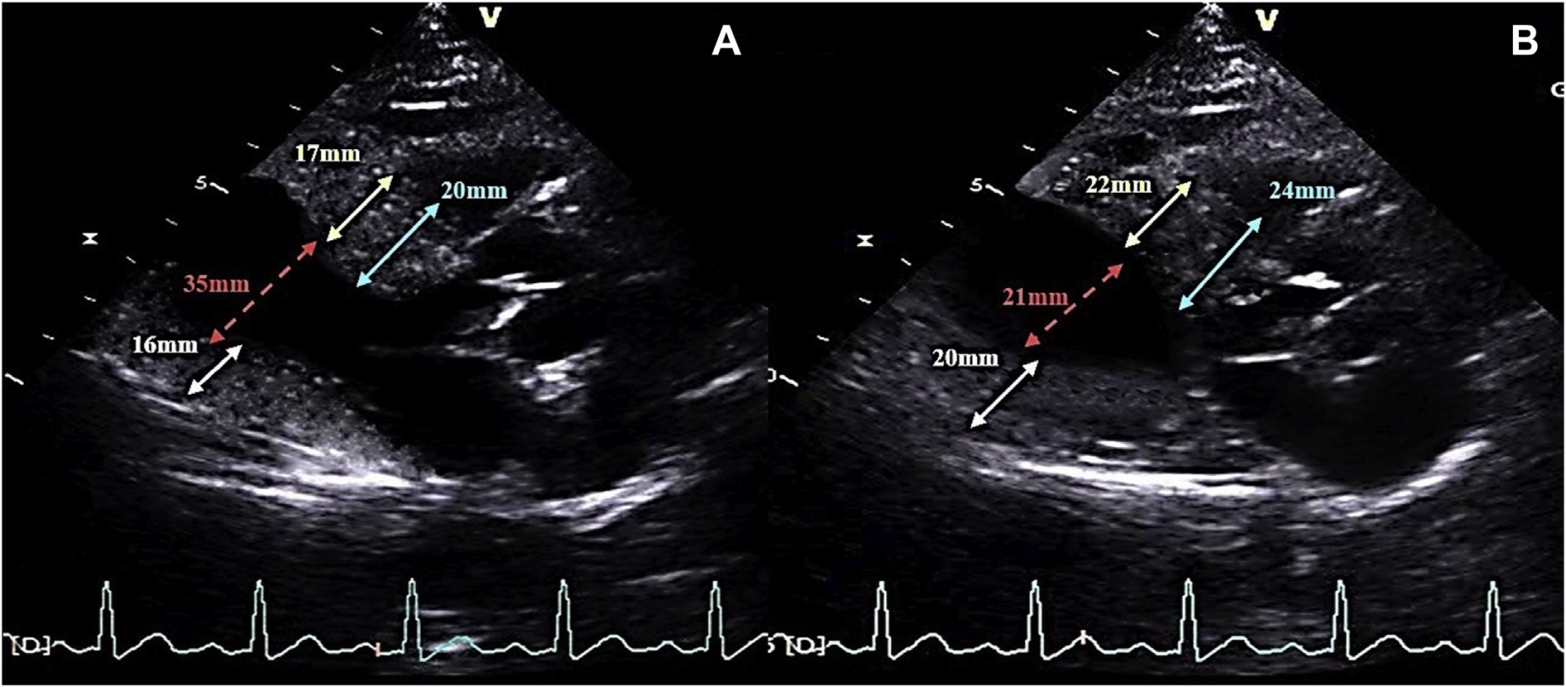
**(A)** Two-dimensional transthoracic echocardiogram (TTE) parasternal long-axis view in diastole showing dimensions of the interventricular septum (17 mm, *yellow arrows*), ventricular cavity (35 mm, *red arrows*), posterior wall (16 mm, *white arrows*), and basal septum (20 mm, *blue arrows*). **(B)** Two-dimensional TTE of parasternal long-axis view in systole showing the dimensions of the interventricular septum (22 mm), ventricular cavity (21 mm), posterior wall (20 mm), and basal septum (24 mm).

**Figure 3:**
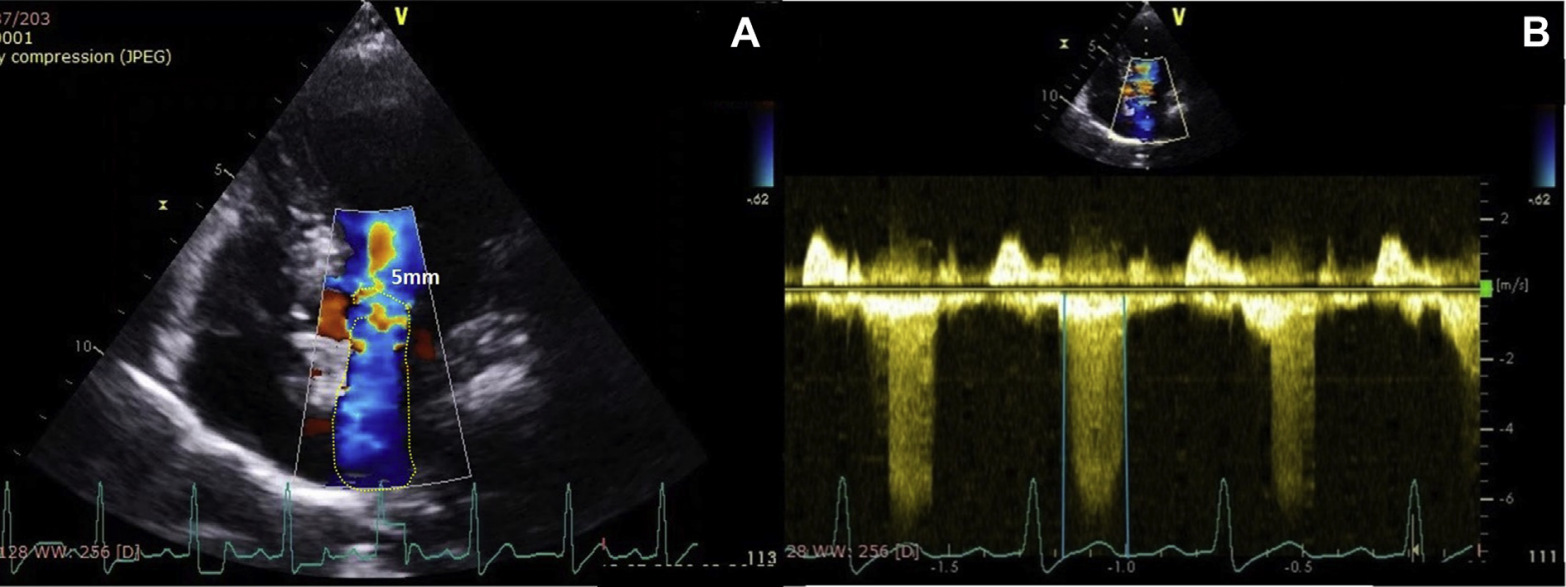
**(A)** Two-dimensional TTE apical four-chamber view with color Doppler evaluation. The mitral regurgitation in mid-late systole was demonstrated to be moderate grade with a regurgitant jet area of 50% and a vena contracta of 5.0 mm **(B)** Two-dimensional TTE with continuous-wave Doppler across the mitral valve showing a mid-late systolic mitral regurgitation (*teallines*) and a peak velocity of 6.1 mm Hg.

**Figure 4:**
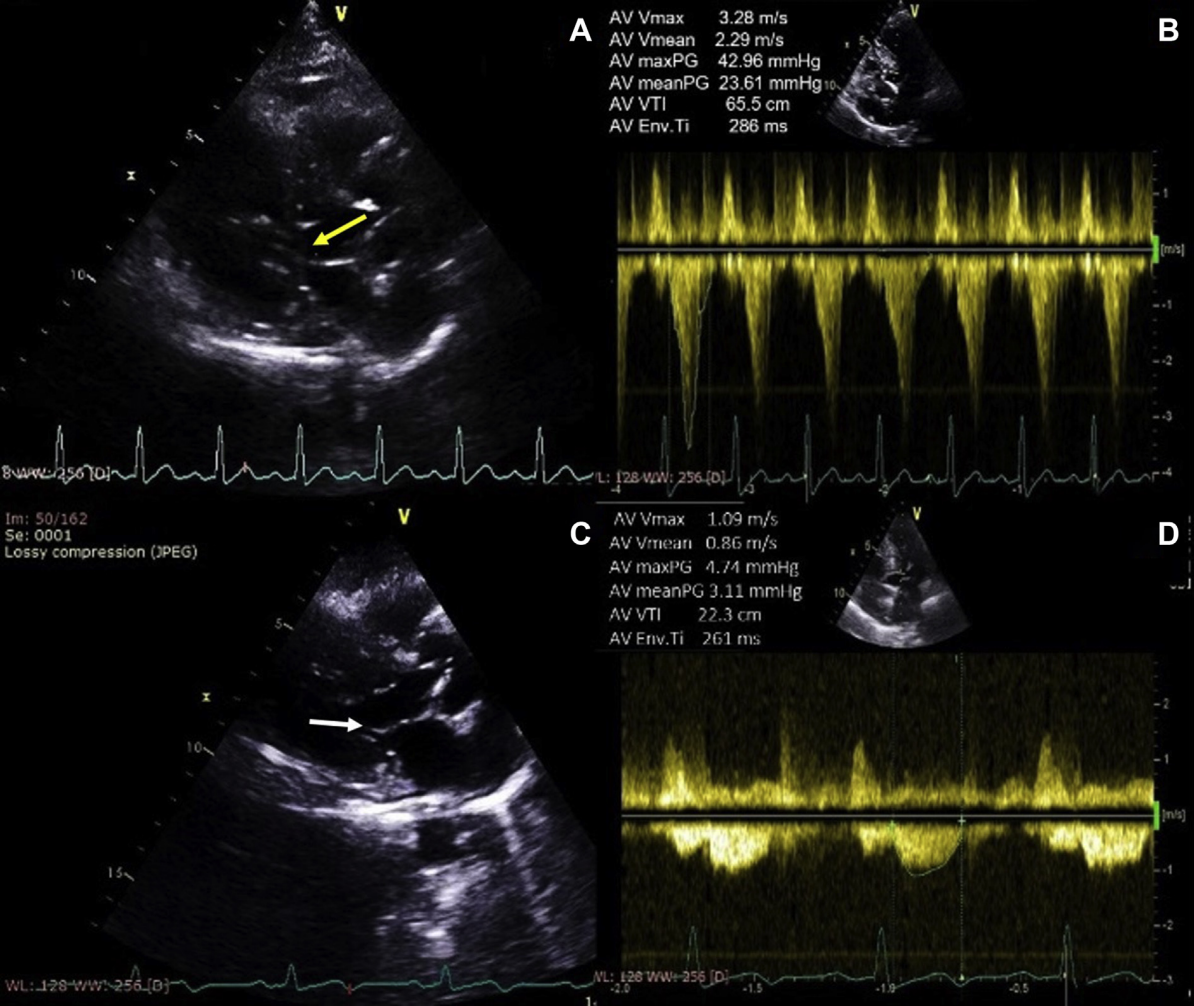
**(A)** Two-dimensional TTE parasternal long-axis view in systole showing systolic anterior motion of the mitral leaflet (*yellow arrow*) approximating with the basal septum. **(B)** Two-dimensional TTE apical 5-chamber view in systole with continuous-wave Doppler across the LVOT showing a late systolic jet with a peak gradient of 43 mm Hg. **(C)** Two-dimensional TTE parasternal long-axis view in systole showing resolution of systolic anterior motion of the mitral leaflet (*white arrow*) after initiating beta-blockers and revascularization. **(D)** Two-dimensional TTE apical 5-chamber view with continuous-wave Doppler across the LVOT showing a reduction of the peak gradient to 5 mm Hg after initiating beta-blockers and revascularization.

**Figure 5:**
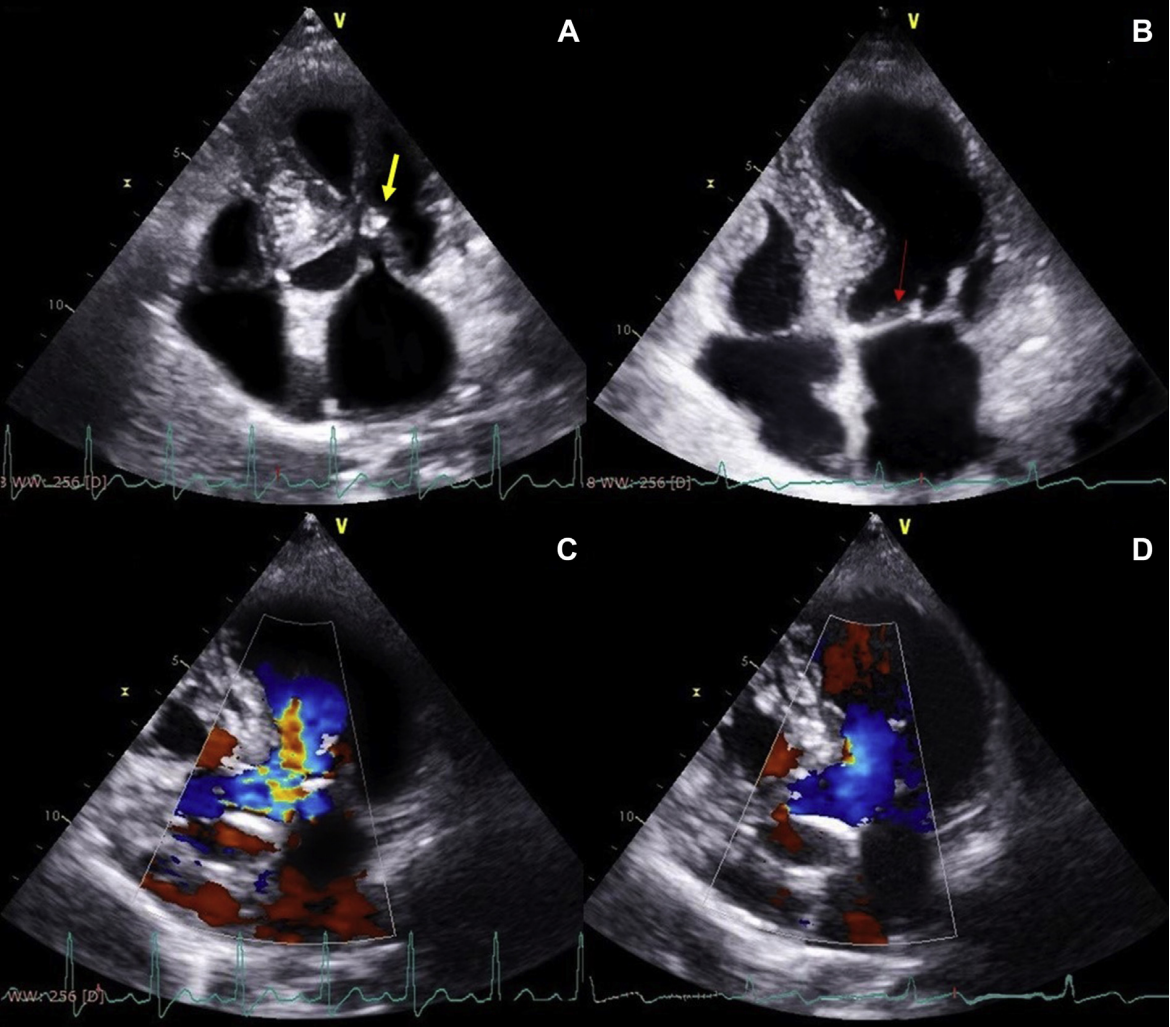
**(A)** Two-dimensional TTE apical four-chamber view showing systolic anterior motion of mitral leaflet (*yellow arrow*) proximate to the basal septum. **(B)** Two-dimensional TTE apical four-chamber view showing resolution of systolic anterior motion of mitral leaflet (*red arrow*) after revascularization. **(C)** Two-dimensional TTE apical five-chamber view with color Doppler evaluation showing turbulence at the LVOT due to dynamic outflow tract obstruction. **(D)** Two-dimensional TTE apical five-chamber view with color Doppler evaluation at LVOT showing resolution of turbulence at LVOT following revascularization.

**Figure 6:**
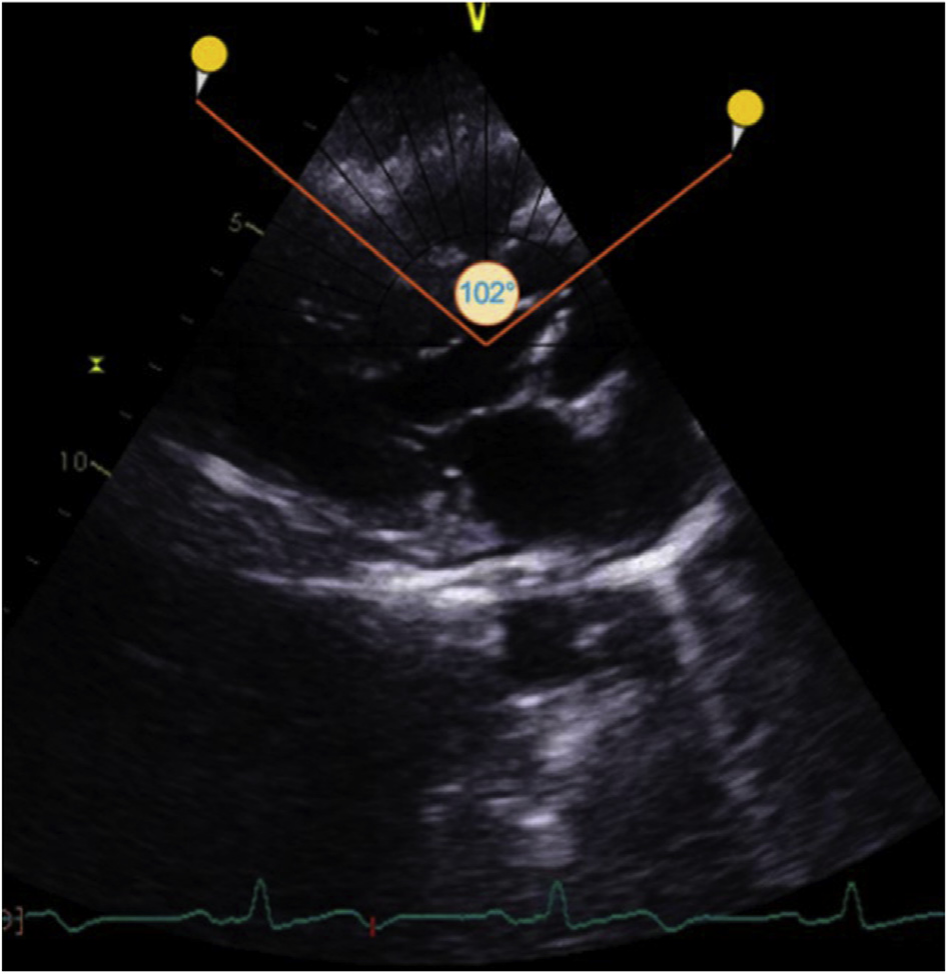
Two-dimensional TTE parasternal long-axis view showing an aortoseptal angle of 102° measured using an online protractor.

**Figure 7:**
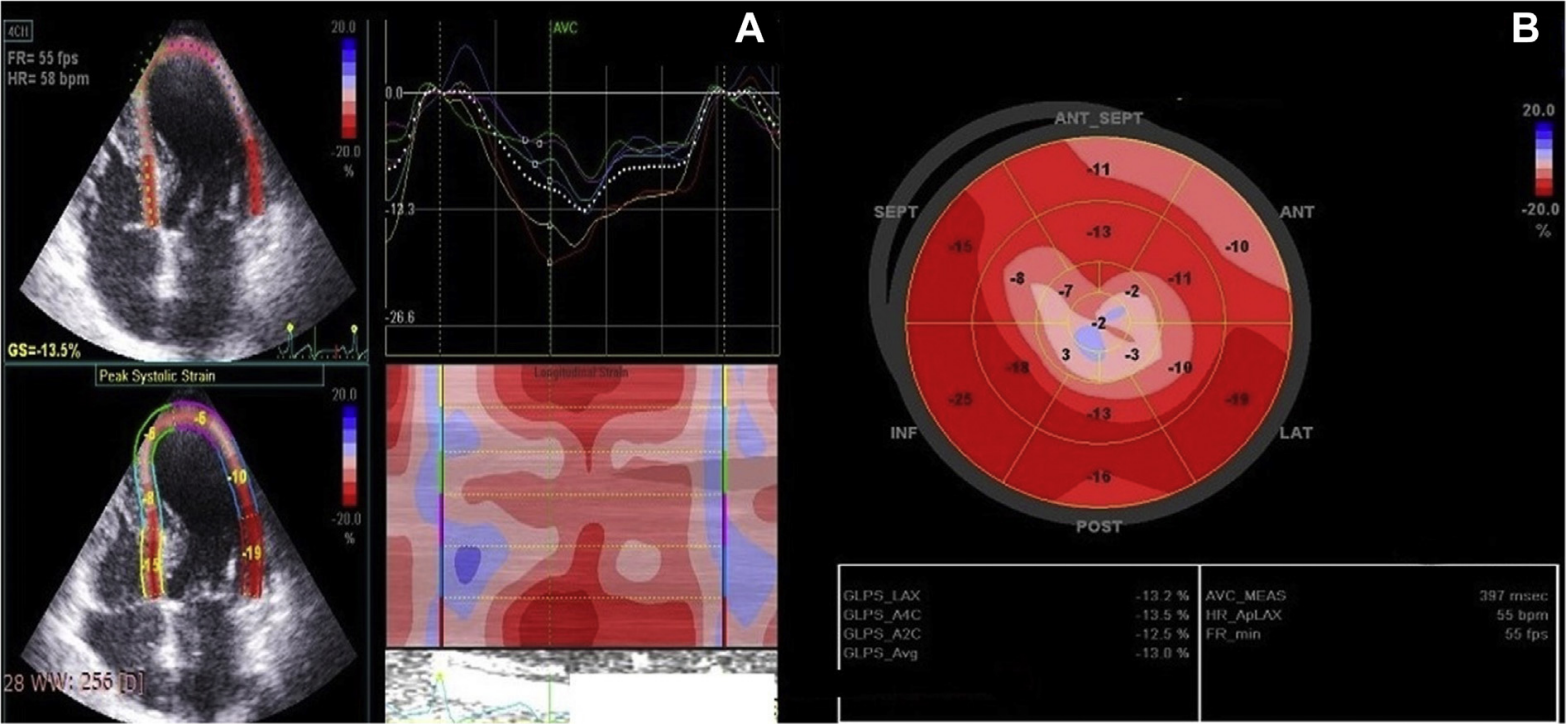
**(A)** Global left ventricular longitudinal strain: quad format display showing postsystolic contraction of the mid and apical segments of the left ventricle with significantly reduced strain in the apex (–6%). **(B)** Bull's-eye plot showing significantly reduced strain in the apical segments and relatively preserved strain in the basal segments, with a global average longitudinal strain of –13%.

Her blood pressure and heart rate during echocardiographic examination were 140/80 mm Hg and 116 bpm, respectively. Her echocardiogram revealed concentric hypertrophy of the left ventricle with septal dimensions of 17 mm at diastole and 22 mm at systole, posterior wall dimension of 16 mm at diastole and 20 mm at systole (Figure 2), estimated left ventricular mass index of 152 g/m^2^ body surface area, and a visually assessed ejection fraction of 70% (Video 1). The basal septum measured 24 mm in systole (Figure 2B). The apical four-chamber view showed a sigmoid-shaped septum with apical hypokinesia and compensatory hyperkinesia of basal segments (Video 2). Color Doppler evaluation at mitral valve showed moderate mitral regurgitation occupying 50% of the left atrium and vena contracta of 5 mm (Figure 3A, Video 3), and continuous-wave Doppler evaluation showed a mid-late systolic jet of mitral regurgitation with a peak velocity of 6.1 m/sec (Figure 3B, Video 3). There was systolic anterior motion of anterior mitral leaflet (Figures 4A and 5A), and color Doppler evaluation showed turbulence at the LVOT (Figure 5C) with a peak velocity of 3.28 m/sec and peak gradient of 43 mm Hg (Figure 4B) by continuous-wave Doppler evaluation. The aortoseptal angle was 102° (Figure 6). Aortic, pulmonary, and tricuspid valve evaluations were normal. Longitudinal strain analysis by speckle-tracking showed postsystolic shortening in the mid and apical segments of the left ventricle with significantly reduced longitudinal strain in the apex (–6%) and relatively preserved strain in the basal septum (–15%; Figure 7A), with a bull's-eye plot corroborating the same (Figure 7B). The patient was started on intravenous metoprolol 2.5 mg (single bolus dose) and an oral regimen of metoprolol tartrate 50 mg twice daily, after which the heart rate was reduced to 64 bpm. The patient underwent fibrinolysis with streptokinase (1.5 million units), and 8 hours after fibrinolysis she underwent pharmacoinvasive angioplasty of the left anterior descending artery with two sirolimus eluting stents of sizes 2.75 × 28 mm and 2.5 × 23 mm. The septal perforator originating from the left anterior descending artery supplying the basal anteroseptum had no flow-limiting lesion that might explain the compensatory hypercontractility of the basal septum (Video 4). Catheter pullback from left ventricular outflow to aorta did not reveal any subaortic or aortic gradients. After initiating beta-blockers (metoprolol tartrate 50 mg twice daily) and revascularization of the left anterior descending artery, the heart rate was reduced to 64 bpm and the intensity of the murmurs decreased substantially. Echocardiographic evaluation after revascularization revealed reduction of gradient at the LVOT from 43 mm Hg to 5 mm Hg (Figure 4D) and resolution of systolic anterior motion of the anterior mitral leaflet (Figures 4C and 5B, Video 5), favoring a possibility of acquired dynamic LVOT obstruction due to acute STEMI.

**Supplemental Figure 1:**
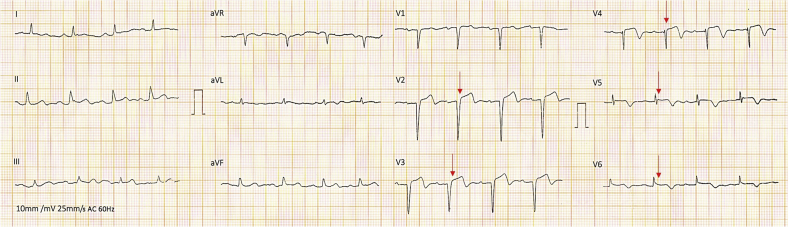
Twelve-lead electrocardiogram taken at the time of readmission due to pulmonary edema showing QS complexes with ST-segment elevation and T inversion (*red arrows*) in precordial leads V2-V6.

Patient was started on guideline-directed medical therapy (aspirin 150 mg once daily, ticagrelor 90 mg twice daily, metoprolol succinate 50 mg twice daily, atorvastatin 80 mg once daily, glimepiride 2 mg once daily, and metformin 1 g twice daily) and discharged uneventfully 4 days after angioplasty. She was advised follow-up echocardiogram 3 months after the procedure with provocative maneuvers (Valsalva/amyl nitrate) or stress echocardiography to determine whether she had hypertrophic cardiomyopathy with LVOT obstruction before the STEMI. This patient discontinued her guideline-directed medical therapy after 1 month and presented 4 weeks later to the emergency department with pulmonary edema. An electrocardiogram was taken that showed QS complexes with ST-segment elevation and T-wave inversion in precordial leads V2-V6 (Supplemental Figure 1). Since the patient was in extremis, we could not perform an echocardiogram in the emergency department. We presume based on clinical presentation and the electrocardiogram that the cause for pulmonary edema could be a reinfarction due to stent thrombosis resulting in heart failure. Resuscitative measures were initiated as per the ACLS protocol (Advanced Cardiac Life Support), which eventually failed, and she succumbed to heart failure.

## Discussion

In a patient presenting with acute myocardial infarction, the causes for a systolic murmur in the apex include acute mitral regurgitation (due to papillary muscle dysfunction or rupture of chordae tendineae) in the left sternal border due to a ventricular septal rupture and acute tricuspid regurgitation (due to right ventricular infarction) in the left third intercostal space due to acute dynamic LVOT obstruction.^4^ Our patient had a high LVOT gradient (peak gradient, 43 mm Hg) with systolic anterior motion of the mitral leaflet. The diagnostic possibilities for an outflow tract gradient and systolic anterior motion of mitral leaflet, namely, hypertrophic cardiomyopathy, hypertensive heart disease, anatomic factors like sigmoid septum and an acute aortoseptal angle leading to obstruction, and an acquired dynamic LVOT obstruction due to myocardial infarction, could be considered. Apical ballooning syndrome (common in postmenopausal women) was ruled out due to a territorial ST-segment elevation (V1-V6 → anteroseptal area of myocardium) in the electrocardiogram and an obstructive lesion in the left anterior descending artery by coronary angiography.

The patient was advised follow-up evaluation with provocative maneuvers (Valsalva/amyl nitrite inhalation) or stress echocardiography^5^ to determine whether she had hypertrophic cardiomyopathy with LVOT obstruction prior to the STEMI, but it could not be performed since she succumbed to heart failure 2 months after revascularization.

As age advances, dilatation and lengthening of the aorta push the septum downward and kink its upper portion, resulting in a more acute angle between the aortic root axis and the interventricular septum.^6^ Additional factors leading to a sharper angulation of the aortic root in elderly individuals include sigmoid-shaped septum and localized hypertrophy of the proximal interventricular septum (ventricular septal bulge).^6^^,^^7^ In normal subjects, the aortoseptal angle varies from 126° to 130°. It has been shown that this angle is smaller in individuals with apparent asymmetric hypertrophy 97° ± 2.6° (mean ± standard error of the mean) than in patients with true asymmetric septal hypertrophy (124° ± 2.9°).^8^ Our patient had an aortoseptal angle of 102° with ventricular septal bulge predisposing to outflow tract obstruction.

The underlying mechanisms of acquired LVOT obstruction in acute STEMI are apical dysfunction and compensatory hypercontractility of the basal segments.^9^ An LVOT obstruction can be precipitated due to left ventricular hypertrophy (due to systemic hypertension); anatomic factors like sigmoid septum and an acute aortoseptal angle^6^; hypercontractility due to stress, anxiety, or inotropic agents such as dobutamine; reduced left ventricular chamber size due to dehydration, bleeding, or diuresis; and intrinsic mitral valve abnormalities like redundant, long anterior leaflet.^10^

The mechanisms leading to reversible dynamic LVOT obstruction in our patient could be categorized into anatomic and hemodynamic factors. Anatomic factors include sigmoid-shaped septum, long-standing hypertension with left ventricular hypertrophy, small left ventricular cavity, and a narrow aortoseptal angle. Hemodynamic factors include tachycardia, anterior wall STEMI resulting in left ventricular apical dysfunction, and compensatory basal hypercontractility leading to obstruction.

Valve gradients are dynamic and vary considerably as a result of changes in heart rate, blood pressure, contractility, and volume status of the individual.^11^ An increase in heart rate increases cardiac contractility (Bowditch staircase phenomenon), which proportionately increases the velocity of blood flow, resulting in a higher pressure gradient across two chambers as governed by Bernoulli's relationship (Δ*P* = 4*V*^2^).^11^^,^^12^ In our patient, with a heart rate of 116 bpm at the time of initial echocardiographic evaluation, the peak gradient was 42.96 mm Hg. For a patient presenting with STEMI, the optimal heart rate to be maintained after treatment with adequate beta-blockers would be 55-65 bpm and the optimal blood pressure would be 110/70 mm Hg. After adequate heart rate control with beta-blockers and revascularization, the heart rate was reduced to 64 bpm with reduction of peak gradient to 4.7 mm Hg.

Since the baseline hemodynamic status of the patient might significantly influence the inflow and outflow gradients, measuring vital parameters, particularly heart rate and blood pressure, is indispensable when performing an echocardiogram.

Treatment of dynamic left ventricular obstruction due to acute STEMI includes adequate intravenous fluids, early beta-blockers to reduce the basal hypercontractility, urgent revascularization to improve apical dysfunction,^10^ and cautious use of inotropes^13^^,^^14^ that might perpetuate outflow tract obstruction.

## Conclusion

This case report highlights a rare mechanical complication of acute myocardial infarction—acquired dynamic LVOT obstruction—the role of an echocardiogram in therapeutic decision-making, the importance of measuring heart rate and blood pressure while performing an echocardiogram, and the role of early beta-blockers and revascularization in relieving dynamic LVOT obstruction.

## Supplementary Data

Video 1 Two-dimensional TTE parasternal long-axis view showing systolic anterior motion of mitral leaflet.

Video 2 Two-dimensional TTE apical four-chamber view showing anterior mitral leaflet proximate to the prominent septal bulge leading to LVOT obstruction.

Video 3 Two-dimensional TTE parasternal long-axis view showing mitral regurgitation.

Video 4 Coronary angiogram showing a discrete 90% stenosis of the proximal segment of left anterior descending artery (*yellow arrow*) and intact septal perforator branch (*orange arrow*).

Video 5 Two-dimensional TTE parasternal long-axis view showing resolution of systolic anterior motion of mitral leaflet following revascularization.
